# Neem leaf glycoprotein prevents post-surgical sarcoma recurrence in Swiss mice by differentially regulating cytotoxic T and myeloid-derived suppressor cells

**DOI:** 10.1371/journal.pone.0175540

**Published:** 2017-04-17

**Authors:** Madhurima Sarkar, Sarbari Ghosh, Avishek Bhuniya, Tithi Ghosh, Ipsita Guha, Subhasis Barik, Jaydip Biswas, Anamika Bose, Rathindranath Baral

**Affiliations:** 1 Department of Immunoregulation and Immunodiagnostics, Chittaranjan National Cancer Institute (CNCI), Kolkata 700026, India; 2 Department of Surgical Oncology and Medical Oncology, Chittaranjan National Cancer Institute (CNCI), Kolkata 700026, India; National Institute of Allergy and Infectious Diseases, UNITED STATES

## Abstract

Post-surgical tumor recurrence is a common problem in cancer treatment. In the present study, the role of neem leaf glycoprotein (NLGP), a novel immunomodulator, in prevention of post-surgical recurrence of solid sarcoma was examined. Data suggest that NLGP prevents tumor recurrence after surgical removal of sarcoma in Swiss mice and increases their tumor-free survival time. In NLGP-treated tumor-free mice, increased cytotoxic CD8^+^ T cells and a decreased population of suppressor cells, especially myeloid-derived suppressor cells (MDSCs) was observed. NLGP-treated CD8^+^ T cells showed greater cytotoxicity towards tumor-derived MDSCs and supernatants from the same CD8^+^ T cell culture caused upregulation of FasR and downregulation of cFLIP in MDSCs. To elucidate the role of CD8^+^ T cells, specifically in association with the downregulation in MDSCs, CD8^+^ T cells were depleted *in vivo* before NLGP immunization in surgically tumor removed mice and tumor recurrence was noted. These mice also exhibited increased MDSCs along with decreased levels of Caspase 3, Caspase 8 and increased cFLIP expression. In conclusion, it can be stated that NLGP, by activating CD8^+^ T cells, down regulates the proportion of MDSCs. Accordingly, suppressive effects of MDSCs on CD8^+^ T cells are minimized and optimum immune surveillance in tumor hosts is maintained to eliminate the residual tumor mass appearing during recurrence.

## Introduction

Surgery is of paramount importance in the management of solid tumors as definitive resection can be curative [1] with chemotherapy and/or radiation therapy or alone. However, post-surgical tumor recurrence in the primary site or in a distant site is a real fact after treatment completion or following a subsequent tumor-free period [2–4]. As recurrence after surgery remains a major cause of morbidity and mortality [5,6], this problem has been addressed by various approaches with a major goal to know the time and location of recurrence, survival of patients with recurrence and to design a treatment modality to prevent tumor recurrence with the ultimate aim to increase patients’ survival [7–12].

In current tumor management, immunotherapy by improvising the host immune system enhances effective tumor clearance [13]. Thus, modulation of a patient’s immune system in such a way after surgery or surgery in combination to chemo/radiotherapy may result in prevention of tumor recurrence. In this context, neem leaf glycoprotein (NLGP), previously reported as a non-toxic immunomodulator to restrict murine tumor growth [14–16], is examined as a post-surgery recurrence preventing agent. NLGP exhibited anti-tumor activity by improving host immunity [17,18] and normalizing angiogenesis [19] in a CD8^+^ T cell-dependent manner, along with decrease in regulatory T cells (Tregs) [20], activation of NK, NKT cells [21], maturation of dendritic cells (DCs) towards DC1 [22] and prevention of conversion of M1 to M2 tumor associated macrophages (TAM) [23]. Evidence suggests that such robust immune modulation not only restricts the tumor growth but also inhibits its metastasis [24].

In clinical settings, regulatory T cells are reported to play an important role in post-surgical tumor recurrence [11,25], but there are few reports stating that the number of MDSCs may indicate the possibility of tumor recurrence, and the role of these cells in initiation and progression of tumor recurrence and how they are regulated during tumor recurrence is not clearly stated [26,27]. These suppressor cells inhibit optimum CD8^+^ T cell functions in an antigen nonspecific way and are primarily mediated by the production of nitric oxide (NO) in combination with a high arginase activity. Arginase 1 activity causes the depletion of arginine and translational blockade of the CD3ξ chain which prevents T cells from responding to various stimuli. High arginase activity in combination with increased NO production by the MDSC results in more pronounced T-cell apoptosis [28–31]. MDSCs crosstalk in initiation and control of tumor recurrence might be a topic of interest.

In this present study, it was demonstrated that NLGP therapy can prevent post-surgical sarcoma recurrence in a CD8^+^ T cell-dependent manner. Furthermore, NLGP-influenced CD8^+^ T cells significantly reduce accumulation and suppressive potential of MDSCs by inducing FAS-mediated cell death, which ultimately favors immune surveillance to maintain the sustained tumor-free state.

## Materials and methods

### Antibodies and reagents

RPMI-1640 and Fetal Bovine Serum (FBS) were purchased from Life Technologies (NY, USA). Lymphocyte separation media (LSM) was procured from MP Biomedicals, Irvine, CA, USA and Hi Media, Mumbai, India. Fluorescence conjugated different anti-mouse antibodies (CD4, Gr1, CD69, CD25-(FITC conjugated) and CD8, CD11b, CD11c, Foxp3, Granzyme B-(PE conjugated)), purified anti-mouse antibodies (CD8, FasR, FasL, IL10, Caspase 3, Caspase 8, cFLIP), Annexin V-PI apoptosis detection kit and IFNγ neutralizing antibody were procured from either BD-Pharmingen or Biolegend (San Diego, CA, USA) or Santa Cruz (Dallas, Texas, USA). Brefeldin A and Concanamycin A were procured from MP Biomedicals, Irvine, CA, USA. LDH cytotoxicity detection kit was purchased from Roche Diagnostics, Mannheim, Germany. Trizol reagent was purchased from Invitrogen (USA) and RevertAid™ first strand cDNA synthesis kit was procured from Fermentas (Waltham, Massachusetts, United States). RT-PCR primers were designed and procured from MWG Biotech AG, Bangalore, India. Western lightining chemiluminescence (Biovision Incorporated, Milipitas, California) and immunoperoxidase color detection (Vector Laboratories Inc, Burlingame, CA94010) kits were purchased. Gr1-DM, CD8-DM particles and BD-IMAG were purchased from BD-Pharmingen (San Diego, CA, USA). Thiopentone sodium [Pentothal Sodium] was purchased from Abbott Laboratories (India) Ltd.

### Mice and tumor inoculation

Female Swiss mice (Age: 4–6 weeks; Body weight: 24–27 g) were obtained from the Institutional Animal Care and Maintenance Department, Chittaranjan National Cancer Institute, Kolkata and maintained in an isolated room under sterile hood (Esco Micro Pite Ltd., Singapore). Autoclaved dry pellet diet (Epic Laboratory Animal Feed, Kalyani, India) and water were given *ad libitum*. Mice were inoculated subcutaneously with peritoneally grown Sarcoma 180 tumor cells (1x10^6^ cells/mice) in the lower right flank to develop solid tumors and a palpable tumor was generally formed after 7–10 days. Tumor area (length x width) and tumor volume (width^2^ x length)/2 were calculated. Tumor bearing mice were euthanized by intra-peritoneal injection of thiopentone sodium (Pentothal Sodium) (@ 100 mg per kg body weight) when tumor size reached 20 mm in either direction or animal looks sick or any necrosis of tumor was occurred. The health of animals was monitored twice a day in every working day and once in holidays. Animal death and abnormal symptoms, if any, were recorded. To minimize animal sufferings, all procedures were performed under thiopentone sodium anesthesia. When it appears that animals were suffering, animals were first separated in special cage with fresh bed and euthanized, if necessary, under supervision of our institutional Veterinary practitioner.

### Neem leaf glycoprotein

An extract from neem (*Azadirachta indica*) leaves was prepared by the method as described previously [32,33]. Matured deep green neem leaves of same size and color (indicative of same age), were collected in summer (April-May) from Salt Lake area of Kolkata, a City of West Bengal, India. These leaves were shed-dried and pulverized. Leaf powder was soaked overnight in phosphate-buffered saline (PBS), pH 7.4. The supernatant was collected by centrifugation at 1500 rpm, extensively dialyzed against PBS, pH 7.4 and concentrated by Centricon membrane filter (Millipore Corporation, MA, USA) with a 10 kDa molecular weight cut-off. The purity of NLGP was checked by Size Exclusion-HPLC (SE-HPLC) in a protein PAK 300 SW column [22]. The protein concentration was measured by Lowry’s method [34]. The biological activity of purified NLGP was checked by tumor growth restriction assay before use.

### Surgical removal of mice solid sarcoma

Tumor surgeries of mice were carried out aseptically after an anesthetization with ether (animals were exposed to ether soaked cotton ball inside a closed chamber for 1–2 min, (ether concentration 1.9% approximately) after i.m. injection of atropine (0.02mg/kg body weight), as described [35]. To maintain proper immune functions and to observe their modulation, we used inhalation anaesthetics rather than injectable one [36] after approval of this justification by Institutional Animal Care and Ethics Committee of CNCI, Kolkata, India. Once unconscious, an incision was made on the right flank (tumor site) using a sterile blade and after visualizing the tumor, it was removed surgically. The site of surgery had sutured using a surgical needle and nylon threads. The area was dabbed with cotton soaked alcohol and covered with an antibiotic powder for a few days. The mice that underwent surgery were kept on a cotton bed under frequent monitoring until the wound healed. All animal experiments, including surgical procedures were performed according to the guidelines established by the Institutional Animal Care and Ethics Committee of CNCI, Kolkata, India, following their approval (Animal Ethical Proposal No. IAEC/1774/RB-3/2015/5). All efforts were made to minimize the suffering of the mice.

### *In vivo* CD8^+^ T cell depletion and PBMC isolation

After surgical removal of solid sarcoma, mice were peritoneally injected with a CD8 depleting antibody (100μg) as demonstrated in Fig 2E. The CD8^+^ T cell depletion status was monitored regularly by analyzing the proportion of CD8^+^ T cells in peripheral blood using flow cytometry. Blood was collected from experimental mice by retro-orbital puncture after ether anesthesia in a heparin coated tube. Mononuclear cells were isolated from heparinized blood using LSM as previously described [17].

### Magnetic assisted cell sorting for MDSCs and CD8^+^ T cells

MDSCs were positively selected from mouse tumor using BD IMag Anti-Mouse Gr1-DM by the manufacturer’s protocol. In brief, cells were incubated with magnetic beads attached to an anti-mouse Gr1 antibody for 30 min in 4^°^C. After incubation, the IMag buffer was added to the cells and was placed in BD Imagnet. After collecting a positive cell fraction, cell purity was checked by flow cytometry and MDSCs with >95% purity was used to check its immunosuppressive properties.

CD8^+^ T cells were purified according to the manufacturer protocol. In brief, PBMCs were incubated with magnetic beads attached to anti-CD8 antibody for 30 min in 4^°^C. After that, the cells were loaded in a MACS column and allowed to pass through. The cellular fraction that stuck to the walls of tubes is the required CD8^+^ T cell fraction. The purity of cells was checked by flow cytometry and cell preparation with >90% purity was taken for experiment. Purified MDSCs’ were co-cultured with CD8^+^ T cells and proliferation of T cells was measured by Ki67 assay as mentioned below. MDSCs with T cell suppressive capacity is used in further assays.

### RNA isolation and RT PCR

Total RNA was isolated using the Trizol reagent. cDNA synthesis was carried out using the RevertAid™ first strand cDNA synthesis kit following the manufacturer's protocol and PCR was carried out using gene-specific oligonucleotide primers, as listed in Table 1. PCR products were identified by image analysis software for gel documentation (Gel Doc™ XR + system, BioRad) following electrophoresis on 1.5% agarose gels, stained with ethidium bromide.

**Table 1:** Primer list*.

**Name**	**Primer sequence (5′–3′)**	**Product Size**
**β Actin F** **β Actin R**	5′ CAACCGTGAAAAGATGACCC 3′ 5′ ATGAGGTAGTCTGTCAGGTC 3′	228bp
**Arginase1 F** **Arginase1 R**	5′ AAGAAAAGGCCGATTCACCT 3′ 5′ CACCTCCTCTGCTGTCTTCC 3′	201bp
**iNOS2 F** **iNOS2 R**	5′ CCTTGTTCAGCTACGCCTTC 3′ 5′ AAGGCCAAACACAGCATACC 3′	203bp
**STAT3 F** **STAT3 R**	5′ GACCCGCCAACAAATTAAGA 3′ 5′ TCGTGGTAAACTGGACACCA 3′	215bp
**TGFβ F** **TGFβ R**	5′ TGCGCTTGCAGAGATTAAAA 3′ 5′ GCTGAATCGAAAGCCCTGTA 3′	197bp
**IL10 F** **IL10 R**	5′ CCAAGCCTTATCGGAAATGA 3′ 5′ TTTTCACAGGGGAGAAATCG 3′	184bp
**MMP9 F** **MMP9 R**	5′ TGAATCAGCTGGCTTTTGTG 3′ 5′ GTGGATAGCTCGGTGGTGTT 3′	242bp
**IDO F** **IDO R**	5′ TGAAAAGCTGCCCACACTGA 3′ 5′ CAGTCCCCACCAGGAAATGA 3′	260bp
**CCL2 F** **CCL2 R**	5′ CTGTGCTGACCCCAAGAAGG 3′ 5′ TGCTTGAGGTGGTTGTGGAA 3′	191bp
**CXCR4 F** **CXCR4 R**	5′ TCAGTGGCTGACCTCCTCTT 3′ 5′ CTTGGCCTCTGACTGTTGGT 3′	203bp
**VEGF F** **VEGF R**	5′ GGACCCTGGCTTTACTGCTG 3′ 5′ CACAGGAAGGCTTGAAGATG 3′	201bp
**IL6 F** **IL6 R**	5′ TTCCATCCAGTTGCCTTCTT 3′ 5′ CAGAATTGCCATTGCACAAC 3′	199bp
**IFNγ F** **IFNγ R**	5′ ACTGGCAAAAGGATGGTGAC 3′ 5′ TGAGCTCAGTGAATGCTTGG 3′	227bp
**Perforin F** **Perforin R**	5′ GATGTGAACCCTAGGCCAGA 3′ 5’ GGTTTTTGTACCAGGCGAAA 3’	161bp
**GranzymeB F** **GranzymeB R**	5’ TCGACCCTACATGGCCTTAC 3’ 5’ TGGGGAATGCATTTTACCAT 3’	198bp
**S100A8 F** **S100A8 R**	5’ GGCCTTGAGCAACCTCATTG 3’ 5’ ATCGCAAGGAACTCCTCGAA 3’	201bp
**S100A9 F** **S100A9 R**	5’ TGGCCAACAAAGCACCTTCT 3’ 5’ TGTGTCCAGGTCCTCCATGA 3’	206bp
**FasR F** **FasR R**	5’ CAGACATGCTGTGGATCTGG 3’ 5’ CATGGTTGACAGCAAAATGG 3’	181bp
**FasL F** **FasL R**	5’ CATCACAACCACTCCCACTG 3’ 5’ GTTCTGCCAGTTCCTTCTGC 3’	152bp
**cFLIP F** **cFLIP R**	5’ TGGGCATGACTACTGTGGAA 3’ 5’ AGGACATGAGTTCCGTGAGG 3’	167bp

*Primers sequences of different genes used in RT-PCR analysis.

### Cell lysis and western blot

PBMC was isolated from blood, and then cells were dissolved in RIPA buffer incubated for 30 min at 4^°^C and centrifuged at 12,000 rpm to collect supernatant as cell-lysate. The protein concentrations of tumor/cell-lysates were determined by Lowry’s method using Folin’s reagent [34]. The tumor lysate or cellular lysate (protein concentration, 30–50 μg) was separated on 12% SDS-PAGE and transferred onto a PVDF membrane (Millipore, USA) using the BioRad Gel Transfer system. The membrane was first blocked with the 5% BSA for 2 hr at room temperature. This was followed by incubation overnight at 4^°^C with the primary antibody, then, with peroxidase-conjugated secondary antibody for 2 hrs at room temperature. Immunoreactive proteins were detected by addition of the HRP color development reagent according to the manufacturer’s protocol and bands were detected by the method described in Refs. [17,18].

### Cytotoxicity assay *in vitro*

The cytotoxicity of T cells (primed with NLGP) against MDSCs purified from solid Sarcoma tumor was tested by LDH release assay using a cytotoxicity detection kit. In brief, the MDSCs purified from tumor were plated as a target in 96-well cell culture plates. The T cells were added in triplicate as effector cells (Effector:Target:10:1) in each well and incubated overnight. In an experiment to find out the role of perforin and FasL in the cytotoxic process, CMA (10 nm/ml) and brefeldin A (10 nm/ml) were used in target effector co-culture. Cell free supernatants were used to measure the level of released LDH using the formula:

$$\%\text{Cytotoxicity}=\frac{\left[\left(\text{Lysis}\;\text{from}\;\text{Effector}+\text{Target}\;\text{Mixture}\right)\text{-Lysis}\;\text{from}\;\text{Effector}\;\text{only}\right]\text{-Spontaneous}\;\text{lysis}}{\text{Maximum}\;\text{lysis-Spontaneous}\;\text{lysis}}\text{x}100$$

### Neutralization of cytokines

For IFNγ neutralization, CD8^+^ T cells from normal mice were purified and were cultured in the presence or absence of NLGP along with/without anti-IFNγ antibody (1 mg/ml). After that, cell free supernatant was collected and used for further experiment.

### Flow cytometric analysis

For flow cytometric analysis, cells (1x10^6^) were stained with fluorescence labeled antibodies (CD8, CD4, CD69, Gr1, CD11b, CD11c, F4/80 and CD25) as per manufacturer’s protocol. After labeling, the cells were washed with FACS buffer (PBS with 1% FBS). Similarly, intracellular molecules such as Perforin, Granzyme B and Foxp3 were stained with anti-mouse fluorescence labeled antibodies using Cytofix-Cytoperm reagents as described before [19,23]. In all flow cytometric staining cells were fixed with 1% para-formaldehyde in PBS and cytometry was performed with Cell Quest software on a FACS Caliber (Becton Dickinson, Mountainview, CA). Suitable negative isotype controls were used to rule out the background fluorescence. The percentage of each positive population and MFI was determined using quadrant statistics. Data was analyzed by either Cell Quest (Beckton Dickinson, Mountainview, CA) or FlowJo software (Tree Star, Ashland, OR).

For flow cytometric staining of Caspase 3, cells were washed with PBS and suspended in 0.25% para-formaldehyde for 15 min at room temperature. These cells were permeabilized with 70% chilled methanol by drop by drop on cells and incubated at 4°C for 60 min. Then, the cells were washed and stained with anti-Caspase 3 antibody for 30 min and the cells were analyzed using flow cytometry as mentioned above.

For AnnexinV-PI staining, the cells were first washed twice with 1ml chilled PBS. Then 1X binding buffer was added to them and they were kept at room temperature for 2 min. Then an equal amount of AnnexinV-PI was added and the cells were kept in the dark for 15–18 min. After addition of 1× binding buffer, the cells were analyzed using flow cytometry.

For flow cytometric analysis of Ki67^+^ proliferating cells, the cells were washed with PBS and then permeabilized using 70% methanol to stain with anti-Ki67 antibody. After labeling with the secondary antibody, the cells were analyzed using flow cytometry.

### Statistical analysis

All results represent the average of 3–5 independent *in vitro* experiments. The number of experiments is stated in the results section and legends to figures. For all assays, a value represents the mean of 3–5 individual observations and is presented as mean ± SD. All pairs of columns were compared using one-way ANOVA with InStat3 software (GraphPad Software, Inc.) with differences between groups attaining a *p* value <0.05 considered as significant.

## Results

### NLGP prevents recurrence of sarcoma after its surgical removal

**Fig 1:**
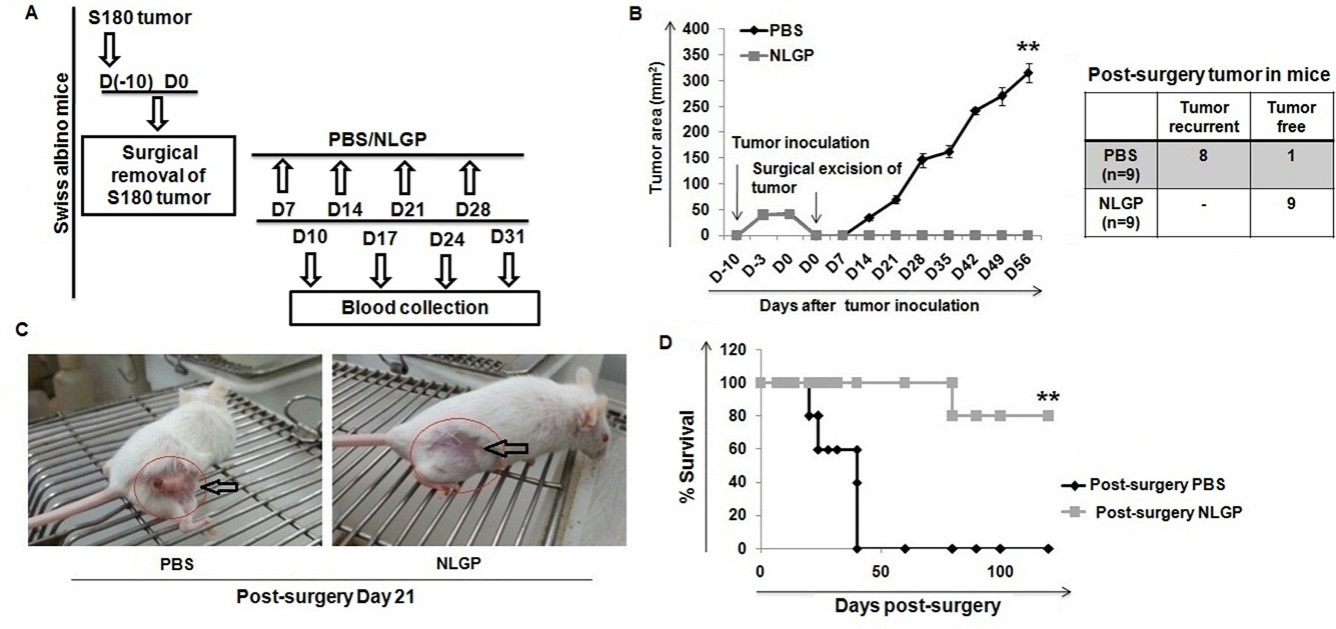
Recurrent tumor growth and survival of Swiss mice with post-surgery NLGP treatment. (A) Experimental design showing sarcoma inoculation, NLGP treatment and blood collection. (B) Recurrent tumor growth curve in pre- and post-surgery phases of mice with or without NLGP treatment (n = 9). (C) Representative photographs of tumor free and tumor bearing mice in the NLGP and PBS groups, respectively, in the post-surgical period. (D) Survival of mice undergoing surgery followed by NLGP treatment (n = 9) (**p<0.001).

Surgical removal of the primary tumor is a useful mode of cancer therapy, but it occasionally suffers by tumor recurrence [1,3]. To mimic the surgical situation experimentally, Swiss albino mice were inoculated with sarcoma cells and then the established tumors (area of the tumors 30–50 mm^2^) were aseptically excised from tumor hosts. On post-surgery day 7, the mice were divided into two groups (n = 9, in each group): the first group of mice was immunized s.c. with NLGP and the other served as control having PBS treatment, once in a week for four weeks till day 30, as outlined in Fig 1A. The mice of both groups were closely monitored for tumor recurrence and mice survival. Within 14–22 days following the surgery, tumor recurrence was noticed in the PBS group of mice with a subsequent steady increase in the tumor size. On the other hand, NLGP immunized mice remained tumor free (Fig 1B) with a comparatively active gesture till day 56 (Surviving mice in PBS group is three till day 56) (Fig 1C). Survivability of mice with surgical removal of tumors from PBS and NLGP was monitored till day 100 (n = 9). The NLGP-treated groups showed significantly greater survivability than the mice having only PBS (Fig 1D).

**Fig 2:**
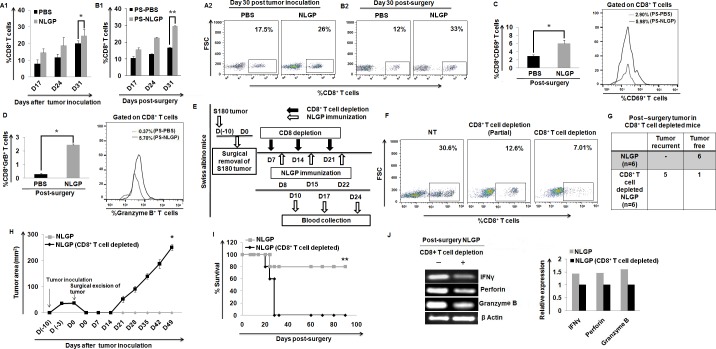
CD8^+^ T cells play an important role in NLGP mediated prevention of tumor recurrence. (A1) Status of CD8^+^ T cells in PBS and NLGP immunized mice after tumor inoculation (n = 9). (B1) Percent positive CD8^+^ T cells in PBS and NLGP immunized mice after surgery (n = 9). A representative figure in both cases is shown in right upper corner panel (A2, B2). (C) Expression of CD69 on CD8^+^ T cells in post-surgical PBS- and NLGP-treated mice (n = 9). (D) Flow cytometric analysis of Granzyme B on CD8^+^ T cells in post-surgical PBS- and NLGP-treated mice (n = 9). Bar diagrams along with representative figures in right panel are shown (C,D). (E) Experimental design showing sarcoma inoculation, CD8^+^ T cell depletion, NLGP immunization and blood collection. (F) Circulating CD8^+^ T cell status following *in vivo* depletion of same cells. (G) Table showing number of recurrent tumor bearing and tumor free mice. (H) Tumor growth curve of recurrent tumor bearing mice in CD8^+^ T cell depleted NLGP immunized mice (n = 6). (I) Survivability curve in NLGP-treated post-surgery mice with or without CD8^+^ T cell depletion (n = 6). (J) RT-PCR analysis of the expression of IFNγ, Perforin and Granzyme B gene expression profile in partial CD8^+^ T cell depleted post-surgery NLGP-treated mice. The bar diagram represents the mean ± SD of three individual observations from each group at each time point (**p<0.001,*p<0.01).

### CD8^+^ T cells in NLGP treated surgically sarcoma removed mice play a crucial role in prevention of recurrence

As tumor recurrence depends on host immunity and NLGP restricts mouse tumor growth in a CD8^+^ T-cell-dependent manner [16,37,38], further the involvement of CD8^+^ T cells in post-surgical prevention of tumor recurrence by NLGP was checked. Assessment of circulating CD8^+^ T cells from the blood of PBS and NLGP immunized mice at different time points was done before and after surgical removal of the mice tumor. In agreement with our earlier reports, it was observed that CD8^+^ T cells increase significantly in NLGP-treated tumor bearing mice (Fig 2A) and also in surgically sarcoma-removed NLGP immunized mice in comparison to PBS controls (Fig 2B). These upregulated CD8^+^ T cells were exhibited as active and cytotoxic, which was evidenced by an increased proportion of CD8^+^CD69^+^ (Fig 2C) and CD8^+^Granzyme B^+^ T cells (Fig 2D).

As the CD8^+^ T cells appear crucial for NLGP-mediated tumor restriction [16] and a post-surgical surge of this particular type of T cells is observed in NLGP-treated mice, it was further ascertained how CD8^+^ T cell depletion affects recurrence in surgically tumor-removed NLGP-treated mice. Swiss mice with palpable sarcoma tumor (n = 12) were operated to remove the tumor mass and, then, one group of mice (n = 6) was depleted for CD8^+^ T cells. On the next day of CD8^+^ T cell depletion, both groups of mice were treated with NLGP as shown in Fig 2E. The depletion of CD8^+^ T cell was confirmed by measuring the circulating CD8^+^ T cells using flow cytometry (Fig 2F). NLGP mediated prevention of recurrence was not observed in the mice where CD8^+^ T cells were depleted (Fig 2G and 2H). CD8^+^ T cell depleted mice survived for a shorter period, than those treated with identical tumor restricting doses of NLGP (Fig 2I). As expected after systemic CD8 depletion, cytotoxic functions of the remaining CD8^+^ T cells were shown to be hampered in CD8 depleted NLGP immunized mice, which correlated well with the downregulated expression of IFNγ, perforin and granzyme B (Fig 2J).

### CD8^+^ T cell depletion mediated tumor recurrence in NLGP-treated mice is predominantly associated with upregulation of Gr1^+^CD11b^+^ MDSCs

**Fig 3:**
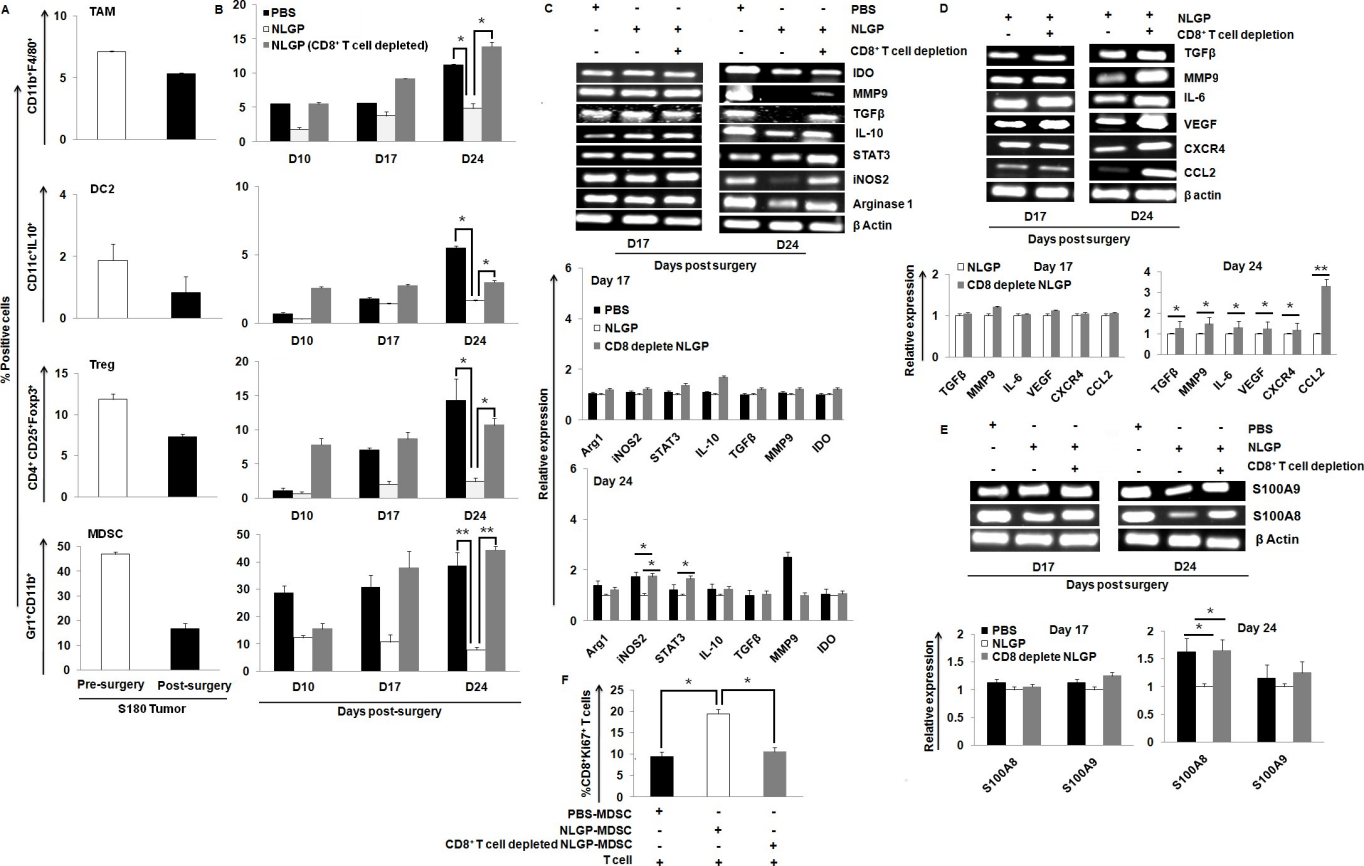
NLGP mediated downregulation of regulatory cells is CD8^+^ T cell dependent. (A) Flow cytometric assessment of the status of TAMs (CD11b^+^F4/80^+^), DC2s (CD11c^+^IL-10^+^), Tregs (CD4^+^CD25^+^Foxp3^+^) and MDSCs (Gr1^+^CD11b^+^) in pre- and post-surgical S180 tumor bearing mice (n = 6). (B) Status of regulatory cells (TAMs, DC2s, Tregs, MDSCs) in post-surgery PBS, NLGP, CD8^+^ T cell depleted NLGP immunized mice (n = 6). (C) RT-PCR analysis to assess the expression of suppressive molecules present in MDSCs in surgically tumor removed PBS, NLGP and CD8^+^ T cell depleted NLGP immunized cohorts (n = 6). (D) Gene expression profile of molecules responsible for MDSC’s differentiation in NLGP and CD8^+^ T cell depleted NLGP immunized surgically tumor removed mice (n = 6). (E) RT-PCR analysis of S100A8 and S1001A9 molecules responsible for MDSCs trafficking in PBS, NLGP and CD8^+^ T cell depleted NLGP immunized surgically tumor removed mice (n = 6). (F) Status of CD8^+^ Ki67^+^ T cells after co culture with MDSCs isolated from PBS, NLGP, CD8+ T cell depleted NLGP mice. Representative figures along with bar diagram showing mean relative expression of three individual mice in each group are presented. (**p<0.001,*p<0.01).

As it was observed that NLGP mediated prevention of tumor recurrence after surgical removal of sarcoma is dependent on CD8^+^ T cells, further the status of different regulatory cells (TAMs, Tregs, DC2, MDSCs) was observed, which may play a rate limiting role on CD8^+^ T cell functions. Accordingly, different immunoregulatory cells were studied within the blood of PBS- and NLGP-treated mice groups after the surgical removal of solid sarcoma. Following removal of the tumor mass, numbers of TAMs, Tregs, DC2 and MDSCs, the ones which were upregulated due to sarcoma load, were significantly downregulated (Fig 3A).

Further the status of regulatory cells from blood of PBS, NLGP treated surgically tumor removed with or without CD8^+^ T cell depletion was checked. It was observed that downregulation was more pronounced in NLGP-treated mice for all cell types especially in case of MDSCs. Downregulated TAMs, Tregs, DC2, MDSCs in NLGP were upregulated, at least in part after CD8^+^ T cell depletion. Prominent upregulation was found in case of MDSCs (Fig 3B).

Above results suggest that NLGP decreases MDSCs in surgically sarcoma removed mice and such decrease in the MDSC number disappeared due to systemic depletion of CD8^+^ T cells. Then, the suppressive nature of these MDSCs from NLGP-treated mice with or without CD8^+^ T cell depletion was observed. In our attempt to check a panel of suppressive molecules in the gene level, it was found more or less downregulation of Arginase 1, iNOS2, STAT3, IL-10, IDO, MMP9 and TGFβ in MDSCs from NLGP-treated surgically sarcoma removed mice. However, depletion of CD8^+^ T cells caused upregulation of these MDSCs’ signature molecules at least in part (Fig 3C), suggesting the regulatory role of CD8^+^ T cells on MDSCs’ suppression.

Interestingly, it was also found increased frequency of MDSCs in CD8 depleted NLGP-treated mice where tumor recurrence occurred. In those mice blood, the status of molecules responsible for MDSCs proliferation, differentiation and trafficking in tumor condition using RT-PCR was examined. Expression of tumor-induced factors responsible for MDSCs’ differentiation and proliferation [39], such as, TGFβ, MMP9, IL-6, VEGF, CXCR4, CCL2 was upregulated in MDSCs from CD8 depleted NLGP immunized mice, that was in downregulation after NLGP treatment (Fig 3D). Again, pro-inflammatory S100 proteins such as S100A8, S100A9 controls the trafficking and accumulation of MDSCs in tumor bearing host [40,41]. Tumor as well as MDSC secret these two proteins which in turn act as an autocrine loop to promote migration of MDSCs within TME. We observed a decrease in the level of S100A8, S100A9 in MDSCs from NLGP-treated mice that were increased on same cells after CD8^+^ T cell depletion (Fig 3E).

To further confirm our *in vivo* observation, an *in vitro* experiment was performed where MDSCs isolated from PBS, NLGP, CD8^+^ T cell depleted NLGP immunized mice were co-cultured with purified CD8^+^ T cells from normal mice. Proliferation of T cells was observed using Ki67 staining. The observed data showed decrease in the proliferation of CD8^+^ T cells where the cells were co-cultured with MDSCs from PBS and CD8^+^ T cell depleted NLGP immunized mice in comparison to those immunized with NLGP (Fig 3F).

### Downregulation of MDSCs in NLGP immunized surgically sarcoma removed mice is associated with CD8^+^ T cell mediated apoptosis through Fas-FasR pathway

**Fig 4:**
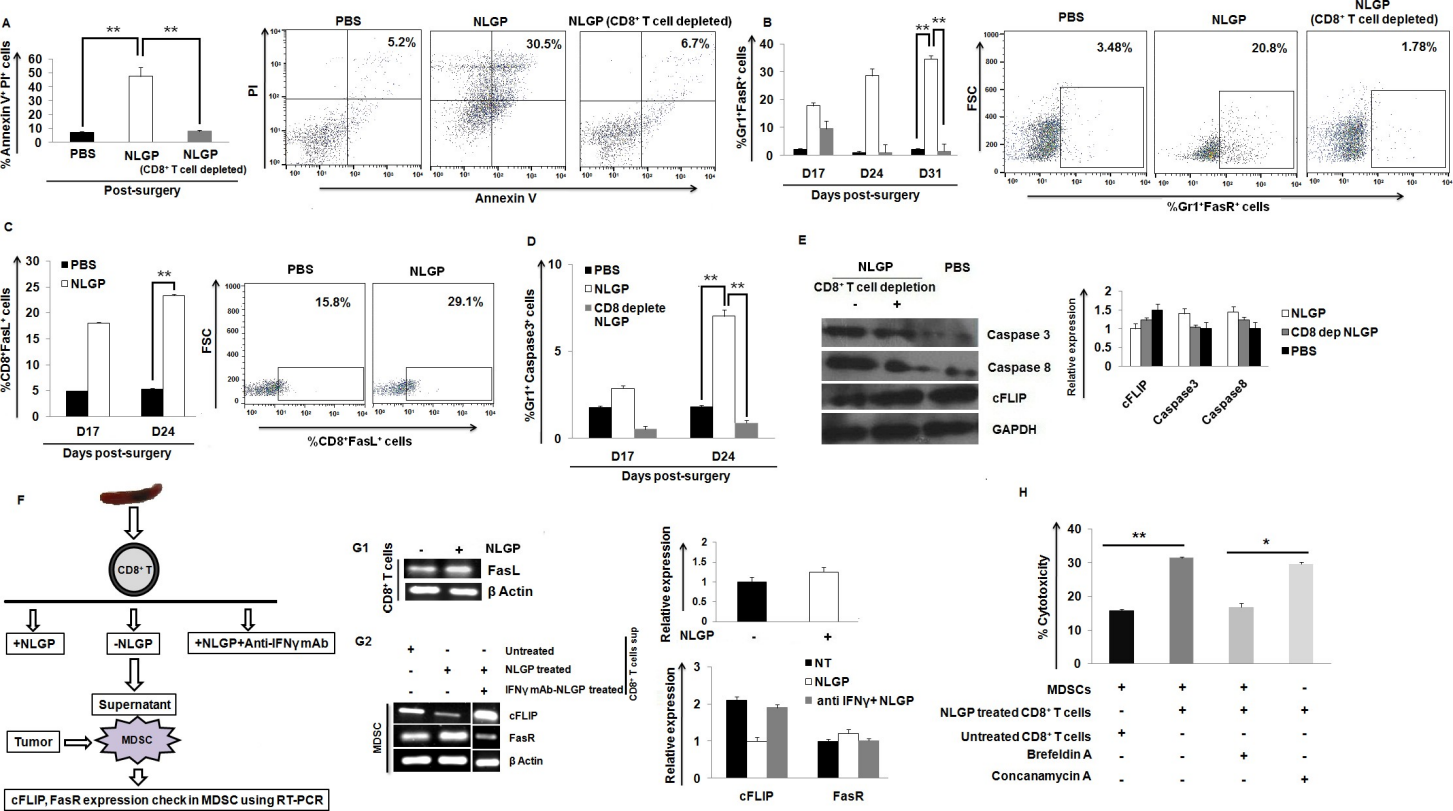
CD8^+^ T cells downregulate MDSCs in Fas dependent pathway. (A) Percentage of Annexin V-PI^+^ MDSCs within the blood of PBS, NLGP, CD8^+^ T cell depleted NLGP immunized mice (n = 6). (B) Flow cytometric assessment of Gr1^+^FasR^+^ MDSCs in post-surgery PBS-, NLGP-treated mice with or without CD8^+^ T cell depletion. (C) Expression of FasL within CD8^+^ T cells in mice with tumor surgery in PBS and NLGP immunized mice. (D) Flow cytometric assessment of Caspase 3 within Gr1^+^ MDSCs in PBS, NLGP and CD8 depleted NLGP immunized mice. (E) Protein level expression of Caspase 3, Caspase 8 and cFLIP within MDSCs from PBS, NLGP and CD8 depleted NLGP immunized surgically tumor removed mice. (n = 6, in each group). (F) Experimental design with MDSCs and CD8^+^ T cells. (G1) Expression of FasL within NLGP-treated CD8^+^ T cells. (G2) Expression of cFLIP and FasR within MDSCs in the presence and absence of supernatants from NLGP-treated CD8^+^ T cells, with or without IFNγ neutralization. (H) Assessment of the cytotoxic potential of NLGP-treated CD8^+^ T cells towards tumor-derived MDSCs, in the presence of Brefeldin A and Concanamycin A. (**p<0.001,*p<0.01). (n = 3, in each group). Bar diagrams along with representative figures are present in each case (A-C).

To confirm the reduction of MDSCs in NLGP immunized mice, the Annexin V-PI^+^ MDSCs in the blood of PBS, NLGP and CD8^+^ T cell depleted NLGP immunized mice was checked. An increased number of apoptotic MDSCs within the NLGP group (Fig 4A) was observed. MDSCs express the death receptor Fas and signals from CD8^+^ T cell expressing FasL caused apoptosis in MDSCs [42–44]. As NLGP treatment decreases pre- and post-surgery MDSC population, and CD8^+^ T cell depletion again ablated such effect, next we have investigated the mechanism of restriction of MDSCs by NLGP-influenced CD8^+^ T cells in our model. Given the importance of Fas-FasL mediated apoptotic signaling in MDSCs, further flow cytometrically the status of FasR on MDSCs and FasL on T cells subsequently in PBS, NLGP and CD8 depleted NLGP immunized mice was assessed. The obtained result clearly suggests that MDSCs from NLGP immunized mice expressed FasR more frequently than PBS-treated mice. However, in CD8^+^ T cell depleted NLGP immunized mice, it was observed that MDSCs lose expression of FasR (Fig 4B). Similarly, FasL status was assessed in CD8^+^ T cells from the blood of PBS, NLGP immunized mice. NLGP treatment results in significantly greater FasL expression on CD8^+^ T cells than PBS-treated controls (Fig 4C). An increased number of Caspase 3^+^ MDSCs was observed in NLGP immunized mice compared to PBS and CD8 depleted NLGP group (Fig 4D). Furthermore, the upregulated expression of caspase 8 and caspase 3 with downregulation of cFLIP within MDSC in NLGP immunized mice was observed in comparison to PBS and CD8 depleted NLGP immunized mice (Fig 4E), which further corroborates the involvement of Fas-mediated killing of MDSCs by NLGP-influenced CD8^+^ T cells. Therefore, NLGP immunization after surgical removal of tumor causes upregulation of CD8^+^ T cells, which in turn apoptose circulating MDSCs, using Fas-FasL mediated pathways.

To further validate the NLGP activated CD8^+^ T cell mediated killing of MDSCs, we *in vitro* cultured purified MDSCs from solid sarcoma in the presence and absence of supernatants of purified CD8^+^ T cells which were treated with NLGP (Fig 4F). After 48 h of culture, MDSCs and CD8^+^ T cells were harvested to isolate RNA. In RT-PCR analysis, an increased FasL expression in NLGP-treated CD8^+^ T cells was observed (Fig 4G1). In the presence of NLGP-treated CD8^+^ T cell culture supernatant, expression of FasR was increased with decrease in the expression of cFLIP in tumor MDSCs in comparison to control group (MDSCs cultured in presence of untreated CD8^+^ T cell supernatant). For further confirmation, IFNγ was neutralized in NLGP-treated CD8^+^ T cells and the purified MDSCs were treated with the collected supernatant from those T cells. It was observed that due to IFNγ neutralization there was no change in the expression of FasR and cFLIP within MDSCs compared to the control group (Fig 4G2).

Finally, the killing efficacy of NLGP stimulated FasL expressing CD8^+^ T cells towards MDSCs was checked by *in vitro* cytotoxicity assay. The purified MDSCs from tumor host were co-cultured with *in vitro* NLGP-treated CD8^+^ T cells and these stimulated T cells kill more number of MDSCs than the CD8^+^ T cells having no stimulation. Again, in the presence of Brefeldin A, a FasL-FasR pathway inhibitor (but not with Concanamycin A, perforin mediated cytotoxicity inhibitor) [45,46], NLGP stimulated CD8^+^ T cell mediated cytotoxicity towards MDSCs was decreased to a significant extent (Fig 4H).

## Discussion

The therapeutic power of NLGP to eradicate solid sarcoma [16] and melanoma [18] was observed. NLGP is a glycoprotein, 33% of it carbohydrate and rest of the part is protein. Both carbohydrate and protein moiety are essential for NLGP’s biological functions, including tumor growth restriction [22]. In context to the therapy of tumor host with NLGP, two practical challenges need to be addressed. Firstly, the management of recurrent tumor loads when the treatment stops, i.e., after completion of 4 weekly NLGP injection schedule. Secondly, the mode of treatment of patients with a large tumor mass. The latter one is the practical problem, as generally cancer patients report to clinic in an advanced disease, when the tumor volume is comparatively large. Again, we observed that NLGP generated central memory T cells are non-functional after its isolation from mice with comparatively higher tumor load (*Ghosh S*. *et al*., *communicated)*. In these perspectives, we have planned to see the power of NLGP to prevent recurrence when the tumor mass is surgically removed.

In such an effort, solid sarcoma tumors (30–50 mm^2^ tumor area) were operated in a group of mice and 50% mice were subsequently treated with NLGP in the post-surgical period to assess the tumor recurrence profile. NLGP-treated mice were recurrence free till day 56 post-surgery. On the other hand, tumor recurrence was observed in all the 9 mice within 15–22 days having vehicle PBS treatment. In our early research, CD8^+^ T cell dependence of NLGP mediated tumor growth restriction was noted [16]. Accordingly, first we checked the CD8^+^ T cell status in the sarcoma bearing host with or without surgical removal of tumor. In line with our previous reports [17], the circulating level of CD8^+^ T cells was high in sarcoma bearing mice treated with NLGP. This enhanced level of CD8^+^ T cells was further increased after removal of tumor in NLGP-treated mice. This initial observation indicates the possible participation of CD8^+^ T cells in NLGP mediated prevention of post-surgical tumor recurrence. Moreover, increase in CD8^+^ T cells after tumor removal suggests the existence of the negative influence of tumor on T cells. These T cells in operated animals are active (CD8^+^CD69^+^) and cytotoxic (CD8^+^GrB^+^), similar to our observation in NLGP-treated tumor hosts. To further confirm the role of CD8^+^ T cells in prevention of post-surgical tumor recurrence, we have taken assistance from CD8^+^ T cell depletion model [19]. Interestingly, NLGP mediated prevention of tumor recurrence after surgery completely disappeared due to CD8^+^ T cell depletion. This establishes the role of these cytotoxic cells in protection of the host from recurred tumors. CD8^+^ T cells chiefly participate in cytotoxic functions, generally mediated by pore formation in targets using perforin and lysis by granzyme B and also by the secretion of higher levels of IFNγ [45]. With depletion of CD8^+^ T cells such cytotoxic functions are diminished or abolished in NLGP-treated mice, which may be one of the reasons of recurrence in the same group of mice.

Immune system is frequently dysregulated in tumor hosts having an array of malignant diseases [47,48]. A tumor itself is the home of several suppressor cells, which are evidenced to have a negative impact on cytotoxic T cell functions [28]. These tumor residing suppressor cells might be involved in suboptimum T cell activity in the tumor bearer. Thus, we first checked the status of predominant suppressor cells, like, TAMs, DC2, Tregs and MDSCs in sarcoma bearing mice before and after surgical tumor removal. Tumor removal resulted in the decrease of these cells in blood that might have direct link to the promoted T cell functions in post-surgery period. Interestingly, in NLGP-treated tumor operated mice such decrease of suppressor cells was more prominent. Among all four types of suppressor cells studied, significant decrease in number was noticed in case of CD11b^+^Gr1^+^ MDSCs. Such downregulation again reciprocally regulated with cytotoxic CD8^+^ T cells. Depletion of CD8^+^ T cells resulted in a proportionate increase of MDSCs, mediating their suppressive functions on different immune cells by implementing co-ordinated function between several biomolecules, like, Arginase1, iNOS2, STAT3, MMP9, etc. Downregulation of MDSCs during the surgical removal of tumor before or after NLGP treatment correlates well with the downregulation of these suppressive molecules. Role of CD8^+^ T cells on MDSCs was also confirmed by the upregulation of these molecules after CD8^+^ T cell depletion. Further evidence suggests the decrease in the concentration of different tumor-derived molecules (e.g., TGFβ, IL-10) because of tumor removal may be involved in the decrease of MDSCs in association with the downregulation in its signature suppressor molecules. NLGP-mediated downregulation of Arginase in TAMs, another suppressor cells, was recently reported [23] and the present result suggests that NLGP by downregulating the suppressor functions of various suppressor cells enhances the cytotoxic functions of CD8^+^ T cells. NLGP also downregulates S100A8, S100A9 in MDSCs to restrict its migration in TME.

We found here the NLGP mediated prevention of tumor recurrence and here, CD8^+^ T cells may participate in removal of suppressor cells, especially MDSCs. In order to find out the mechanism of decrease of MDSCs, we have checked the cytotoxic potential of NLGP stimulated CD8^+^ T cells on MDSCs *in vitro*, as it is difficult to demonstrate in *in vivo* in multicellular environment of tumor. It was demonstrated clearly that NLGP-treated CD8^+^ T cells can kill a greater proportion of suppressive MDSCs than untreated CD8^+^ T cells. Regarding the mechanism of cytotoxicity of MDSCs by T cells, we have seen upregulated FasL expression on NLGP-treated CD8^+^ T cells rather than control. MDSCs are expressive of FasR that was seen to be upregulated *in vitro in* presence of IFNγ secreting NLGP-treated CD8^+^ T cells supernatant. For further confirmation, we have designed an *in vitro* experiment where CD8^+^ T cells were treated with NLGP, and IFNγ was neutralized in these CD8^+^ T cell culture. In presence of supernatant from these T cell culture, FasR expression was decreased in MDSCs of IFNγ neutralized group compare to NLGP-treated group. Confirmation of participation of FasR-FasL pathway in cytotoxicity was obtained by blocking Fas pathway in CD8^+^ T cell-MDSC co-culture in presence of Brefeldin A. Blocking of FasR–FasL pathway resulted downregulation of CD8^+^ T cell mediated cytotoxicty of MDSCs.

In conclusion, the results suggested possible mechanism of prevention of post-surgery sarcoma recurrence by NLGP by maintaining surgery mediated decrease of MDSCs. With assistance from CD8^+^ T cell depletion model, it was evidenced that NLGP by activating CD8^+^ T cells downregulate the proportion of MDSCs that helps in maintenance of optimum immune surveillance in tumor hosts to eliminate the residual tumor mass during recurrence.

## Supporting information

S1 File Raw data and graph of all results. (XLS)

S2 File Tumor growth (in mm^3^) curve. Tumor growth curves of PBS vs NLGP and NLGP vs CD8^+^ T cell depleted + NLGP groups of mice in day dependent manner are given.
(XLS)

## Abbreviations

PBS: Phosphate buffered saline
NLGP: Neem leaf glycoprotein
S180: Sarcoma 180
MDSCs: Myeloid derived suppressor cells
DC: Dendritic cells
TAM: Tumor associated macrophages
